# Health disparities influence peripheral venous access insertion time in the emergency department: An observational study

**DOI:** 10.1371/journal.pone.0336171

**Published:** 2025-12-26

**Authors:** Charlotte O’Sullivan, Nicholas Mielke, Yuying Xing, Amit Bahl

**Affiliations:** 1 Oakland University William Beaumont School of Medicine, Rochester, Michigan, United States of America; 2 Department of Medicine, Creighton University School of Medicine, Omaha, Nebraska, United States of America; 3 Corewell Health Research Institute, Royal Oak, Michigan, United States of America; 4 Department of Emergency Medicine, Corewell Health William Beaumont University Hospital, Royal Oak, Michigan, United States of America; NYU Grossman School of Medicine: New York University School of Medicine, UNITED STATES OF AMERICA

## Abstract

**Objective:**

This retrospective, multicenter study aimed to investigate disparities in peripheral intravenous catheter (PIVC) placement wait times among patients in emergency departments (EDs), focusing on the impact of factors such as race, sex, age, and comorbidities.

**Methods:**

Electronical health record (EHR) data from four EDs within Corewell Health System were analyzed for adult patients who underwent PIVC placement between January 1st, 2021 and January 31st, 2023. Multivariable linear regression models were employed to analyze associations between patient demographics (including race, sex, age, and comorbidities) and PIVC placement wait times. Adjustments were made for Charlson comorbidity index, emergency severity index, hospital size, obesity, and method of PIVC insertion.

**Results:**

Among 319,938 PIVC placements analyzed, significant disparities were observed: Black patients waited 9.65% longer for PIVC placement compared to White patients (p < 0.001). Women experienced a 6.67% longer wait time than men (p < 0.001). Obese and elderly patients also experienced prolonged wait times. These disparities persisted across both ultrasound-guided and traditionally placed PIVCs.

**Conclusions:**

This study underscores substantial disparities in PIVC placement wait times in EDs, influenced by race, sex, age, and comorbidities. Addressing these disparities is crucial for improving equity in emergency care delivery. Future research should focus on implementing targeted interventions to mitigate these disparities and enhance timely access to essential medical interventions for all patient populations.

## Introduction

In 2021, the United States had approximately 139.8 million emergency department (ED) visits [1]. Unfortunately, the quality of care received in the ED is impacted by patient factors such as sex, race, and age, resulting in healthcare disparities [2–4]. For example, women are less likely to be examined within an hour of presenting to the ED with chest pain compared to men even when comorbidities are adjusted for and possibly due to sex based bias at presentation [5]. Additionally, there is evidence of bias based on sex, race, and ethnicity when patients are triaged in the ED [6]. Data shows that Black patients were less likely to be prioritized in rooming compared to White patients with similar triage scores. [3].Additionally, Black men and women were less likely to receive electrocardiograms, oxygen saturation measurements, and chest radiographs in the workup of chest pain than non-Black men [7]. Age also impacts care, and it has been shown that older patients wait longer for imaging in the ED compared to younger patients [8].

One unifying characteristic of ED care is the usage of peripheral intravenous catheters (PIVCs). About 75% of ED and 90% of hospitalized patients require peripheral vascular access for diagnostics, medications, fluids, or blood products [9–14]. In the ED setting specifically, early and reliable vascular access is crucial for the administration of these potentially life-saving therapies. For example, the emergent management of acute ischemic stroke requires rapid intravenous thrombolysis. Studies show that even modest reductions in door-to-needle time—by as little as 15 minutes—significantly improve patient outcomes [15,16]. Similarly, prompt vascular access is critical in the treatment of sepsis, where delays in administering intravenous fluids and antibiotics are associated with increased mortality [17]. Even though PIVC access is a routine procedure, the failure rates of PIVC placement are reported to range from 36% to 63% [11,12,18–20].

In a recently published large multicenter investigation of over 140,000 hospitalizations, Black patients and female patients were predictors of PIVC complications and failure in hospitalized patients [21]. However, outcomes related to PIVC placement were not reported in the analysis and generally remain understudied in the ED. Given that PIVC access is a foundational procedure for initiating a range of workups and therapies, we aim to determine the impact of health disparities on the time required to establish functional PIVC access.

## Methods

### Study design, Setting, and Participants

This study was an observational multicenter investigation that utilized electronic health record (EHR) data. The inclusion criteria were: adult (over age 18) patients who underwent PIVC placement during their emergency encounter at any of four of Corewell Health System’s emergency departments (William Beaumont University Hospital, Troy, Farmington Hills, or Grosse Pointe) between January 1^st^, 2021, and January 31^st^, 2023. For each encounter, epidemiological, demographic, therapeutic, clinical, and outcomes data were collected. The exclusion criteria included patients with missing PIVC placement documentation.

### Ethical Statement

This study was approved by the Corewell Health Institutional Review Board (IRB 2023−097) on July 12, 2023. A waiver for the requirement of informed consent was granted due to its retrospective design. All data were fully anonymized before accessed, and authors did not access identifying information during data collect. All data were accessed between November 9^th^ and December 31^st^, 2023.

### Study Definitions

As defined by the National Institute on Minority Health and Health Disparities, a health disparity is a health difference based on one or more health outcomes that adversely affect disadvantaged populations [22]. Racial and ethnic minority groups, as well as sexual and gender minority groups, are all included as disadvantaged populations. Based on the United States Centers for Disease Control and Prevention definition, body mass index was categorized as underweight, healthy weight, overweight, or obesity [23]. ICD10 codes were queried for each encounter’s past medical history, problem list, and discharge diagnosis (excluding the index encounter). EDs stratify the urgency of a patient’s presenting symptoms using the Emergency Severity Index (ESI) [24]. The Infusion Nurses Society Standards of Practice was used to define vesicant and/or irritant use [25].

### Data sources/measurement

The electronic health record system, Epic Systems (Verona, Wisconsin, United States) was the data source.

### Outcomes and Measurements

The primary outcome was PIVC wait time. PIVC wait time was the time in minutes between the patient registering in the ED at arrival and receiving PIVC placement. The secondary outcome was PIVC wait time by mode of placement: ultrasound-guided or traditional.

### Statistical Analysis

Descriptive analyses were conducted to summarize patient characteristics. Continuous variables were summarized using means, standard deviations, medians, and interquartile ranges. Categorical variables were presented as frequencies and percentages. The ANOVA test was used to compare continuous variables across groups, while the Chi-square test was used for categorical variables. Multivariable linear regression was used to evaluate the relationships between demographic variables and PIVC placement wait time, controlling for confounding variables identified through clinical knowledge. These confounders included the Charlson comorbidity index, ESI, hospital size, obesity, and insertion method. Due to skewness from some extended IV wait times, PIVC placement wait time was log-transformed (LN) for analysis. Back-transformed results were expressed as percentages for reporting purposes. All results were reported with corresponding 95% confidence intervals (CIs) and p-values. Statistical significance was defined as a p-value less than 0.05, and all tests were two-sided. Analyses were performed using R-4.3.1 (R Foundation for Statistical Computing).

## Results

There were 319,938 PIVCs placed in the ED between January 1^st^, 2021, and January 31^st^, 2023, that met the inclusion criteria. The average age was 56 years old, and 59.11% were female. The median duration from ED arrival to PIVC placement was 1.28 hours (interquartile range [IQR] 0.72–2.29). The majority of patients identified as White (60.27%), followed by Black (33.09%), Asian (2.17%), American Indian or Alaskan Native (0.33%), or Other (4.14%). PIVCs were most commonly 20 gauge (n = 245,237, 79.96%) placed in the antecubital fossa (n = 206,129, 64.43%) using the traditional palpation technique (308,186, 96.33%). (Table 1)

**Table 1:** Demographics, hospital course, PIVC characteristics, and outcomes of emergency department patients. For continuous variables, medians (interquartile ranges, IQRs) and means (standard deviation, SD) were presented. For categorical variables, frequencies (percentage) were presented. Abbreviations: ED = Emergency Department, PIVC = peripheral venous catheter.

		Duration from ED Arrival to PIVC Placement				
Variables^‡^	All	<0.72 hours (1st Quartile)	0.72–1.27 hours (2nd Quartile)	1.28–2.29 hours (3rd Quartile)	≥ 2.30 hours (4th Quartile)	*p* value
n	319,938	82587 (25.8%)	78810 (24.6%)	78761 (24.6%)	79780 (24.9%)	
**Demographics**						
Age, category						<0.001
18-64	199574 (62.38%)	50695 (61.38%)	50379 (63.92%)	49457 (62.79%)	49043 (61.47%)	
65-80	77561 (24.24%)	20539 (24.87%)	18302 (23.22%)	18644 (23.67%)	20076 (25.16%)	
80+	42803 (13.38%)	11353 (13.75%)	10129 (12.85%)	10660 (13.53%)	10661 (13.36%)	
Age, years						<0.001
Mean	55.78 (20.65)	56.22 (20.78)	54.82 (20.84)	55.53 (20.77)	56.51 (20.14)	
Median	57.00 (38.00, 72.00)	58.00 (38.00, 73.00)	55.00 (36.00, 72.00)	56.00 (37.00, 72.00)	57.00 (39.00, 73.00)	
Sex						<0.001
Male	130818 (40.89%)	37166 (45.00%)	32519 (41.26%)	30943 (39.29%)	30190 (37.84%)	
Female	189120 (59.11%)	45421 (55.00%)	46291 (58.74%)	47818 (60.71%)	49590 (62.16%)	
Race						<0.001
White	192828 (60.27%)	50071 (60.63%)	48286 (61.27%)	48501 (61.58%)	45970 (57.62%)	
Black	105860 (33.09%)	27313 (33.07%)	25175 (31.94%)	24792 (31.48%)	28580 (35.82%)	
Asian	6951 (2.17%)	1704 (2.06%)	1724 (2.19%)	1813 (2.30%)	1710 (2.14%)	
American Indian or Alaska Native	1060 (0.33%)	241 (0.29%)	248 (0.31%)	274 (0.35%)	297 (0.37%)	
Other	13239 (4.14%)	3258 (3.94%)	3377 (4.28%)	3381 (4.29%)	3223 (4.04%)	
Body mass index, kg/m2						<0.001
Mean	29.55 (8.10)	29.10 (7.90)	29.41 (7.90)	29.62 (8.12)	30.07 (8.43)	
Median	28.15 (23.86, 33.64)	27.68 (23.63, 33.00)	28.06 (23.83, 33.45)	28.25 (23.92, 33.67)	28.49 (24.14, 34.33)	
Body mass index, category						<0.001
Underweight	10475 (3.27%)	3038 (3.68%)	2477 (3.14%)	2494 (3.17%)	2466 (3.09%)	
Healthy weight	89700 (28.04%)	24304 (29.43%)	22396 (28.42%)	21880 (27.78%)	21120 (26.47%)	
Overweight	91896 (28.72%)	24057 (29.13%)	22901 (29.06%)	22561 (28.64%)	22377 (28.05%)	
Obesity	127867 (39.97%)	31188 (37.76%)	31036 (39.38%)	31826 (40.41%)	33817 (42.39%)	
Charlson Comorbidity Index						<0.001
0	150543 (47.05%)	38946 (47.16%)	38386 (48.71%)	37251 (47.30%)	35960 (45.07%)	
1-2	59740 (18.67%)	15732 (19.05%)	14068 (17.85%)	14364 (18.24%)	15576 (19.52%)	
3-4	24755 (7.74%)	6595 (7.99%)	5648 (7.17%)	6007 (7.63%)	6505 (8.15%)	
>=5	12354 (3.86%)	3128 (3.79%)	2965 (3.76%)	3106 (3.94%)	3155 (3.95%)	
Not documented	72546 (22.68%)	18186 (22.02%)	17743 (22.51%)	18033 (22.90%)	18584 (23.29%)	
Hospital Course						
Hospital Setting						<0.001
Community (250 + beds)	54098 (16.91%)	14608 (17.69%)	15821 (20.07%)	12466 (15.83%)	11203 (14.04%)	
Community (300 + beds)	48989 (15.31%)	18051 (21.86%)	10167 (12.90%)	9267 (11.77%)	11504 (14.42%)	
Mid-size (500 + beds)	107323 (33.54%)	20906 (25.31%)	30055 (38.14%)	31301 (39.74%)	25061 (31.41%)	
Academic (1000 + beds)	109528 (34.23%)	29022 (35.14%)	22767 (28.89%)	25727 (32.66%)	32012 (40.13%)	
Emergency severity index						<0.001
Mean	2.77 (0.58)	2.57 (0.61)	2.79 (0.57)	2.83 (0.56)	2.89 (0.50)	
Median	3.00 (2.00, 3.00)	3.00 (2.00, 3.00)	3.00 (2.00, 3.00)	3.00 (3.00, 3.00)	3.00 (3.00, 3.00)	
Use of intravenous vesicant						<0.001
Yes	36269 (11.34%)	10916 (13.22%)	8484 (10.77%)	8427 (10.70%)	8442 (10.58%)	
Length of stay, hours						<0.001
Mean	59.27 (108.78)	65.39 (116.14)	54.10 (103.93)	56.25 (105.66)	61.00 (108.28)	
Median	13.12 (4.92, 74.05)	24.08 (4.27, 79.53)	8.12 (4.13, 69.30)	9.25 (4.77, 72.08)	15.81 (6.72, 75.05)	
Disposition from ED						<0.001
Admission	124871 (39.03%)	37023 (44.83%)	28729 (36.45%)	29190 (37.06%)	29929 (37.51%)	
Discharge	195067 (60.97%)	45564 (55.17%)	50081 (63.55%)	49571 (62.94%)	49851 (62.49%)	
PIVC Characteristics						
Gauge						<0.001
18	42668 (13.91%)	18151 (22.78%)	9256 (12.15%)	7914 (10.48%)	7347 (9.76%)	
20	245237 (79.96%)	58481 (73.39%)	62138 (81.55%)	62326 (82.51%)	62292 (82.75%)	
22	17963 (5.86%)	2937 (3.69%)	4600 (6.04%)	5040 (6.67%)	5386 (7.16%)	
24	818 (0.27%)	111 (0.14%)	201 (0.26%)	257 (0.34%)	249 (0.33%)	
Not documented	13252	2907	2615	3224	4506	
Orientation						<0.001
Left	154042 (48.80%)	41982 (51.40%)	37485 (48.14%)	36949 (47.60%)	37626 (47.93%)	
Right	161615 (51.20%)	39688 (48.60%)	40377 (51.86%)	40681 (52.40%)	40869 (52.07%)	
Not documented	4281	917	948	1131	1285	
Location						<0.001
Antecubital	206129 (64.43%)	54970 (66.56%)	51863 (65.81%)	50556 (64.19%)	48740 (61.09%)	
Forearm	70471 (22.03%)	17395 (21.06%)	16879 (21.42%)	17334 (22.01%)	18863 (23.64%)	
Hand/Wrist	8218 (2.57%)	2188 (2.65%)	1926 (2.44%)	2063 (2.62%)	2041 (2.56%)	
Upper Arm	10456 (3.27%)	1958 (2.37%)	2154 (2.73%)	2661 (3.38%)	3683 (4.62%)	
Other	24664 (7.71%)	6076 (7.36%)	5988 (7.60%)	6147 (7.80%)	6453 (8.09%)	
Insertion Method						<0.001
Traditional	308186 (96.33%)	81420 (98.59%)	76959 (97.65%)	75426 (95.77%)	74381 (93.23%)	
Ultrasound Guided	11752 (3.67%)	1167 (1.41%)	1851 (2.35%)	3335 (4.23%)	5399 (6.77%)	
Outcomes						
ED Arrival to PIVC Placement, hours						<0.001
Mean	1.74 (1.50)	0.43 (0.19)	0.99 (0.16)	1.73 (0.29)	3.85 (1.45)	
Median	1.28 (0.72, 2.30)	0.45 (0.28, 0.60)	0.98 (0.85, 1.13)	1.70 (1.48, 1.97)	3.40 (2.77, 4.50)	
Dwell time, hours						<0.001
Mean	32.00 (46.12)	34.72 (47.31)	30.07 (44.75)	30.76 (45.31)	32.30 (46.87)	
Median	7.68 (3.30, 46.27)	12.15 (3.65, 49.68)	6.42 (3.18, 44.07)	6.70 (3.20, 44.93)	7.93 (3.22, 45.62)	
Proportion of Dwell to Hospital Length of Stay						<0.001
Mean	0.71 (0.28)	0.79 (0.28)	0.76 (0.26)	0.70 (0.27)	0.60 (0.28)	
Median	0.79 (0.52, 0.97)	0.91 (0.73, 0.99)	0.81 (0.65, 0.98)	0.72 (0.53, 0.97)	0.58 (0.37, 0.88)	
Removal Reason						<0.001
Completed By Discharge	170991 (57.01%)	41820 (54.48%)	43256 (58.25%)	43082 (58.15%)	42833 (57.24%)	
Failure	44935 (14.98%)	12848 (16.74%)	10246 (13.80%)	10634 (14.35%)	11207 (14.98%)	
Therapy Completed	84007 (28.01%)	22093 (28.78%)	20756 (27.95%)	20373 (27.50%)	20785 (27.78%)	
Not documented	20005	5826	4552	4672	4955	

A multivariable linear regression model was performed. After adjusting for Charlson comorbidity index, ESI, hospital size, obesity, and insertion method, females waited 6.7% longer than males for PIVC placement (p < 0.001). Obese patients waited 6.8% longer than patients with a healthy weight (p < 0.001). Patients over age 65 waited 3.6% to 4.4% longer than patients between the ages of 18–64 (p < 0.001). Compared to patients who identified as White, Black patients waited 9.6% longer (p < 0.001), American Indians or Alaska Natives waited 6.5% longer (p = 0.019), and Asian patients waited 2.3% longer (p = 0.0356). (Table 2).

**Table 2:** Multivariable Linear Model of PIVC Wait Time in Total Population. For continuous variables, medians (interquartile ranges, IQRs) and means (standard deviation, SD) were presented. For categorical variables, frequencies (percentage) were presented.

Terms^‡^	Estimate Percentage	Estimate	p value
Demographics			
Age, category			
18-64	Reference	Reference	
65-80	4.4%	0.0432 (0.0357, 0.0508)	<0.001
> 80	3.6%	0.0354 (0.0257, 0.0452)	<0.001
Sex			
Male	Reference	Reference	
Female	6.7%	0.0646 (0.0584, 0.0708)	<0.001
Race			
White	Reference	Reference	
Black	9.6%	0.0921 (0.0847, 0.0996)	0
Asian	2.3%	0.0224 (0.0015, 0.0433)	0.0356
American Indian Or Alaska Native	6.5%	0.0629 (0.0103, 0.1154)	0.019
Other	0.6%	0.0064 (−0.009, 0.0218)	0.4151
Body mass index, category			
Healthy weight	Reference	Reference	
Underweight	−1.0%	−0.0101 (−0.0278, 0.0075)	0.2606
Overweight	2.9%	0.0286 (0.0206, 0.0367)	<0.001
Obesity	6.8%	0.0656 (0.0581, 0.0732)	<0.001
Charlson Comorbidity Index			
0	Reference	Reference	
1-2	3.0%	0.03 (0.0216, 0.0384)	<0.001
3-4	4.0%	0.039 (0.0269, 0.051)	<0.001
≥ 5	7.0%	0.0677 (0.0515, 0.0839)	<0.001
Not available	0.0%	−0.0002 (−0.0081, 0.0077)	0.9587
Emergency Severity Index	50.6%	0.4096 (0.4042, 0.415)	<0.001
Hospital			
Beaumont Farmington Hills	Reference	Reference	
Beaumont Grosse Pointe	−8.4%	−0.0873 (−0.0981, −0.0766)	<0.001
Beaumont Royal Oak	28.0%	0.2468 (0.2374, 0.2561)	<0.001
Beaumont Troy	22.9%	0.2058 (0.1959, 0.2156)	<0.001
Insertion Method			
Traditional	Reference	Reference	
Ultrasound Guided	64.8%	0.4996 (0.4833, 0.5158)	<0.001

Among PIVCs placed under ultrasound-guidance (n = 11,752), a multivariable linear model was performed and adjusted for the the Charlson comorbidity index, ESI, hospital size, and obesity. It demonstrated that females waited 8.9% longer than males for ultrasound-guided PIVC placement (p < 0.001). Obese patients waited 6.1% longer than patients with a healthy weight (p < 0.001). Patients between 65 and 80 years old waited 9.2% longer than patients between the ages of 18 and 64 (p < 0.001), while patients over 80 years old waited 4.3% longer (p = 0.045). Compared to patients who identified as White, Black patients waited 7.4% longer (p < 0.001), American Indians or Alaska Natives waited 29.33% longer (p = 0.0192), and Asian patients did not wait significantly longer (p > 0.05). (Table 3).

**Table 3:** Multivariable Linear Model of PIVC Wait Time in Ultrasound Guided PIVC Population. For continuous variables, medians (interquartile ranges, IQRs) and means (standard deviation, SD) were presented. For categorical variables, frequencies (percentage) were presented.

Terms^‡^	Estimate Percentage	Estimate	p value
Demographics			
Age, category			
18-64	Reference	Reference	
65-80	9.2%	0.0881 (0.0577, 0.1184)	<0.001
> 80	4.3%	0.0422 (0.0009, 0.0836)	0.0453
Sex			
Male	Reference	Reference	
Female	8.9%	0.0849 (0.0561, 0.1137)	<0.001
Race			
White	Reference	Reference	
Black	7.4%	0.0716 (0.0416, 0.1017)	<0.001
Asian	−3.4%	−0.0343 (−0.1565, 0.0879)	0.582
American Indian Or Alaska Native	29.3%	0.2572 (0.0419, 0.4726)	0.0192
Other	−6.6%	−0.0679 (−0.1617, 0.0258)	0.1555
Body mass index, category			
Healthy weight	Reference	Reference	
Underweight	−4.0%	−0.0411 (−0.1237, 0.0416)	0.33
Overweight	1.2%	0.0117 (−0.0264, 0.0498)	0.5468
Obesity	6.1%	0.0594 (0.0257, 0.0931)	0.0006
Charlson Comorbidity Index			
0	Reference	Reference	
1-2	2.6%	0.0259 (−0.0072, 0.0589)	0.1248
3-4	2.3%	0.0232 (−0.0163, 0.0626)	0.2497
≥ 5	1.8%	0.0181 (−0.0305, 0.0666)	0.4656
Not available	−3.8%	−0.039 (−0.0881, 0.0101)	0.1197
Emergency Severity Index	54.1%	0.4322 (0.4088, 0.4557)	<0.001
Hospital			
Beaumont Farmington Hills	Reference	Reference	
Beaumont Grosse Pointe	−12.6%	−0.1342 (−0.1821, −0.0864)	<0.001
Beaumont Royal Oak	45.7%	0.3765 (0.3361, 0.417)	<0.001
Beaumont Troy	14.1%	0.1317 (0.0866, 0.1768)	<0.001

## Discussion

This multicenter observational study of over 300,000 PIVCs placed in the ED is one of the first to examine the relationship between health disparities and time to establish a PIVC. Our findings reveal inequities in PIVC wait times that persist even after adjusting for baseline comorbidities and severity of illness at ED presentation. Females experienced a 6.7% longer wait time for PIVC placement compared to males, and Black patients waited 9.6% longer than their White counterparts. Additionally, adults over age 65 experienced at least a 4% increase in wait time. These findings not only highlight the prevalence of disparities in vascular access but also point to systemic gaps that disproportionately impact vulnerable patient populations.

Disparities in vascular access were especially pronounced among elderly, female, Black patients, and obese patients when considering the classification of Difficult Venous Access (DIVA) [19]. This group, which comprises up to 59.3% of hospitalized patients, represents the most challenging cases of vascular access and often necessitates advanced visualization technologies such as ultrasound [25–27]. Biological differences likely exacerbate these challenges—women often have smaller veins, individuals with darker skin tones may have less visible veins, and obesity is a well-documented risk factor for DIVA [28–31]. These factors, combined with limited resources, skills, and clinician shortages, inherently complicate vascular access and perpetuate inequities in care delivery which may also be present due to structural and interpersonal bias and stereotyping [32].

Efforts to address these challenges have included early escalation to ultrasound-guided PIVC placement, as recommended by recently updated clinical practice guidelines [26,27]. While this approach has improved success rates in some instances, it also places increased demands on healthcare resources and workforce capacity. Many institutions lack adequately trained clinicians to perform ultrasound-guided PIVC placements efficiently, leading to longer wait times for DIVA patients [32]. Unfortunately, these delays further amplify the inequities experienced by vulnerable groups, exacerbating disparities in patient outcomes.

Our study brings to the forefront a critical issue affecting disadvantaged populations. The prolonged wait times observed suggest that these groups are more likely to undergo multiple placement attempts, resulting in higher rates of needlesticks and patient discomfort. Despite advancements in vascular access methods, these findings underscore the pressing need for targeted interventions, increased workforce education, and continued research to address the underlying disparities. Achieving equitable access to vascular care is essential to improving outcomes for all patients, particularly those who face systemic barriers to timely and effective treatment.

## Limitations

This study has several limitations. First, as a retrospective analysis using EHR data, the accuracy and completeness of documentation may vary, potentially introducing bias. However, the large sample size and inclusion of multiple hospital sites help mitigate this concern. Second, unmeasured confounders such as ED staffing levels, patient throughput, and departmental workflow were not accounted for, which may have influenced PIVC placement wait times. Third, race and ethnicity were categorized into broad groups, which may not fully capture the nuances of individual identity or the intersection of multiple racial and ethnic backgrounds. Despite these limitations, the findings provide valuable insights into disparities in vascular access and highlight the need for further investigation.

## Conclusions

This study reveals persistent disparities in PIVC placement wait times, disproportionately affecting Black patients, women, the elderly, and those with obesity. Addressing these inequities requires targeted interventions to improve workflow efficiency and vascular access training. Future efforts should focus on ensuring equitable, timely care for all patients.
